# Optimizing interleukin-2 concentration, seeding density and bead-to-cell ratio of T-cell expansion for adoptive immunotherapy

**DOI:** 10.1186/s12865-021-00435-7

**Published:** 2021-07-03

**Authors:** Sasan Ghaffari, Monireh Torabi-Rahvar, Sajjad Aghayan, Zahra Jabbarpour, Kobra Moradzadeh, Azadeh Omidkhoda, Naser Ahmadbeigi

**Affiliations:** 1 Gene Therapy Research Center, Digestive Disease Research Institute, Tehran University of Medical Sciences, Tehran, Iran; 2 Department of Hematology, School of Allied Medical Sciences, Tehran University of Medical Sciences, Tehran, Iran; 3 Department of Immunology, School of Medicine, Tehran University of Medical Sciences, Tehran, Iran

**Keywords:** T-lymphocyte, Interleukin-2, Immunotherapy, Primary cell culture

## Abstract

**Background:**

The successful ex vivo expansion of T-cells in great numbers is the cornerstone of adoptive cell therapy. We aimed to achieve the most optimal T-cell expansion condition by comparing the expansion of T-cells at various seeding densities, IL-2 concentrations, and bead-to-cell ratios. we first expanded the peripheral blood mononuclear cells (PBMCs) of a healthy donor at a range of 20 to 500 IU/mL IL-2 concentrations, 125 × 10^3^ to 1.5 × 10^6^ cell/mL, and 1:10 to 10:1 B:C (Bead-to-cell) ratios and compared the results. We then expanded the PBMC of three healthy donors using the optimized conditions and examined the growth kinetics. On day 28, CD3, CD4, and CD8 expression of the cell populations were analyzed by flow cytometry.

**Results:**

T-cells of the first donor showed greater expansion results in IL-2 concentrations higher than 50 IU/mL compared to 20 IU/mL (*P* = 0.02). A seeding density of 250 × 10^3^ cell/mL was superior to higher or lower densities in expanding T-cells (*P* = 0.025). Also, we witnessed a direct correlation between the B:C ratio and T-cell expansion, in which, in 5:1 and 10:1 B:C ratios T-cell significantly expanded more than lower B:C ratios. The results of PBMC expansions of three healthy donors were similar in growth kinetics. In the optimized condition, 96–98% of the lymphocyte population expressed CD3. While the majority of these cells expressed CD8, the mean expression of CD4 in the donors was 19.3, 16.5, and 20.4%.

**Conclusions:**

Our methodology demonstrates an optimized culture condition for the production of large quantities of polyclonal T-cells, which could be useful for future clinical and research studies.

## Background

Adoptive immunotherapy is currently an attractive approach to cancer treatment. It is defined as the application of expanded/manipulated immune cells adoptively transferred to a cancer patient to eliminate tumor cells, which can be used as a stand-alone treatment or in combination with traditional methods of radiotherapy, chemotherapy, and surgery [1, 2]. One of the interesting modalities of adoptive immunotherapy is the adoptive transfer of T cells as the key player of anti-tumor immunity [3, 4]. The three T cells of interest in this approach include tumor-infiltrating lymphocytes, T cell receptor T cells (TCR T cells), and chimeric antigen receptor T cells (CAR T cells), the latter of which has sparked greater attention among researchers and clinicians [5, 6]. To deliver an effective adoptive T cell transfer, adequate expansion of cells is an essential step. This may not be an issue for hematological malignancies in which remission is not closely correlated with the number of T cells. However, T cell dose is particularly important in solid tumors where, in some cases, a few hundred billion T cells are required [7]. Accordingly, an efficient T cell expansion method to produce such numbers is a prerequisite of a successful ACT.

Most ACT protocols include isolating and in vitro expanding peripheral mononuclear cells (PBMCs). In order to produce polyclonal T cells, resting T cells must first be activated using a primary and secondary signal through CD3 and CD28 stimulation, respectively. T cell activation can be achieved using anti-CD3 antibodies (OKT3) and irradiated feeder cells; however, a more accessible strategy to activate T cells is simply adding small magnetic beads coated with anti-CD3/CD28 antibodies [8]. Beads are substitutes for antigen-presenting cells (APCs) and are one of three activation methods approved for clinical trials, which can be coated with any antibodies, are simple to use and remove, and can be used to separate any T cell subset [9]. The number of beads to cells, called bead-to-cell ratio, is a determinant of signal strength and consequently T cell expansion. While extremely high bead-to-cell ratio results in T cell exhaustion, too low ratios fail to activate T cells, both resulting in a suboptimal T cell expansion [10]. After activation, cells are seeded in culture plates. The number of cells seeded per mL is another determinant of effective cell expansion outcome, which has been previously shown in studies using osteoblasts, mesenchymal and adipose stem cells, and human umbilical vein endothelial cells [11–14]. For T cells, however, the optimized condition of successful expansion has not been clearly defined.

T cells require growth factors to expand, which are catered by the medium and serum. One growth factor is interleukin-2 (IL-2), which by binding to its receptor promotes the polyclonal expansion of T cells through binding to its cell surface receptor. Subsequently, the complex is quickly internalized, and then the α subunit (CD25) resurfaces to bind to IL-2. Hence, in an expansion course, it is pivotal to replenish IL-2 in the culture medium every 2–3 days [15]. Studies have used a wide range of 20 to 7200 IU/mL of IL-2 concentration for T cell expansion [16], though their difference in expansion efficacy is not clear.

Despite the improvement of T cell expansion protocols over the years, the optimum conditions for expansion are still elusive since there are many determinant factors. Hence, this study was designed to investigate the optimum condition for polyclonal T cell expansion with regards to three essential factors, i.e., IL-2 concentration, seeding density, and bead-to-cell (B:C) ratio.

## Results

### Morphological observations

**Fig. 1:**
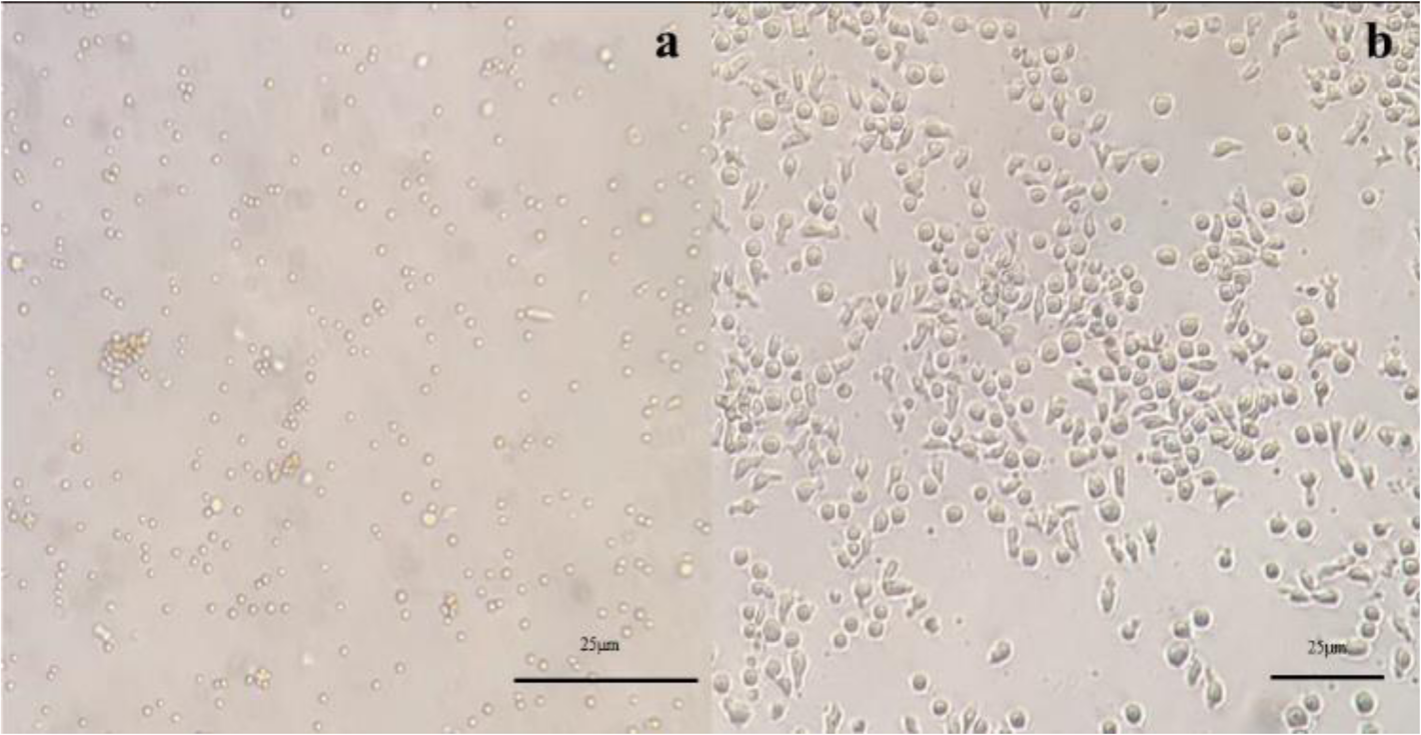
The microscopic view of PBMCs on day 0 (**a**) and the expanded T cells on day 3 (**b**). After activation with anti-CD2/CD3/CD28 coated beads, cell sizes considerably increased

Within the first 2 h of culture, cells started to form clusters of cells. This was evident in either low- and high-density wells or low and high B:C ratios; however, it was clear that the higher the density or the B:C ratio was the more aggregates were observed. Since the cells’ capacity to proliferate diminishes after 21 days and the beads are not replenished after day 0, cells fail to form clusters in the final weeks. We also observed an increase in the size of mononuclear cells 3–4 days after the addition of beads (Fig. 1). This was seen in both the microscopic view and the forward and side scatter plots of T cells in flow cytometry.

### The effect of IL-2 on T cell expansion

**Fig. 2:**
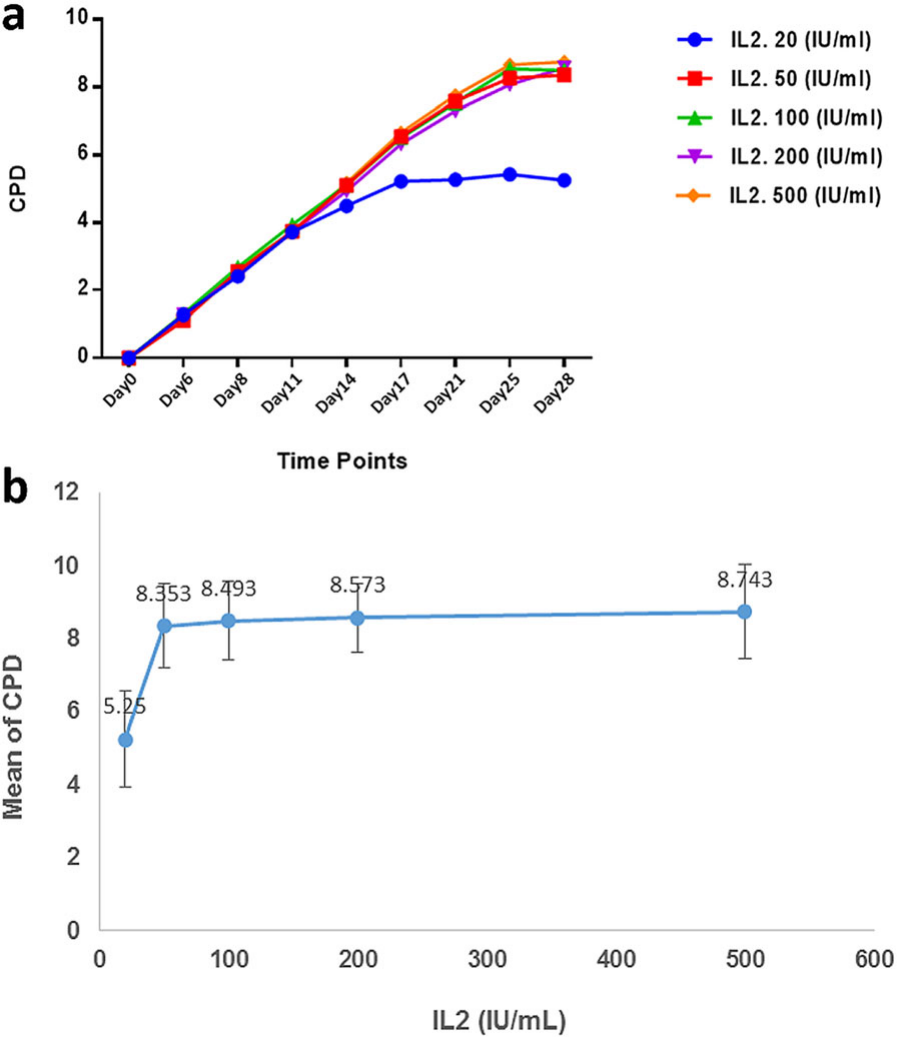
CPD of T cells after expansion with different IL-2 concentrations. The expansion trend in (**a**) shows increased expansion in higher than 20 IU/mL concentrations. On day 28, there was no significant difference between 50, 100, 200, and 500 IU/mL concentrations (**b**)

Five groups were assigned to determine the effect of varying IL-2 concentrations on T cell expansion. Cells supplemented with 20 IU/mL of IL-2 had the lowest expansion rate compared to other groups, which was statistically significant. When the IL-2 concentration increased to 50 IU/mL, the expansion rate significantly increased in comparison to 20 IU/mL (*p* = 0.02). Although a further increase in IL-2 concentration resulted in more expansion rate, on day 28, there was no difference between cells cultivated in 500 IU/mL compared to 50 (*p* = 0.7), 100 (*p* = 0.8), and 200 IU/mL (*p* = 0.84) (Fig. 2).

### The effect of seeding density on T cell expansion

**Fig. 3:**
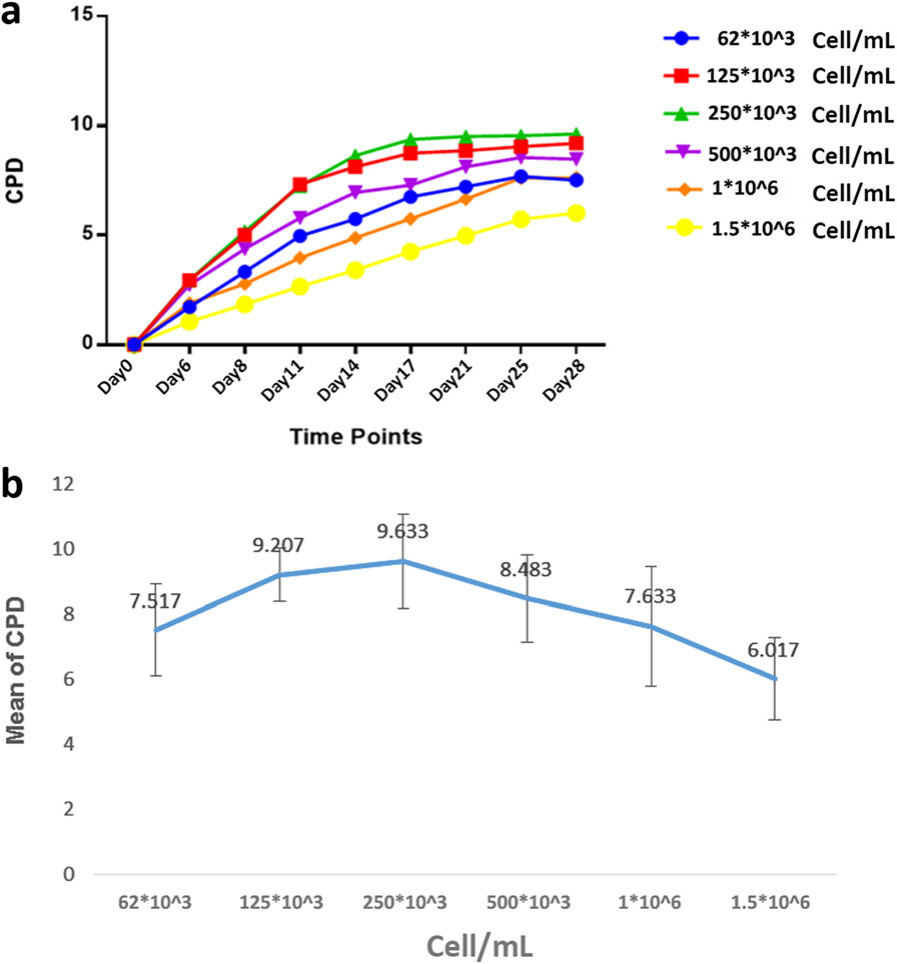
CPD of T cells when cultured in various cell densities. Graph (**a**) compares the trend of expansion in different groups. On day 28, the highest and lowest expansion rates occurred in 250 × 10^3^ and 1.5 × 10^6^ cell/mL, respectively (**b**)

T cells expanded in different cell densities showed diverse results. The cells seeded at 250 × 10^3^ cell/mL and cells seeded at 1.5 × 10^6^ cell/mL led to the highest and lowest expansion rates, respectively. An increase in the number of seeded cells from 62 × 10^3^ cell/mL to 250 × 10^3^ cell/mL gradually improved expansion rate; however, further increases resulted in lower T cell expansions. On day 28, cells seeded at 250 × 10^3^ cell/mL resulted in significantly higher fold expansions compared with seeding densities of 62 × 10^3^ (*p* = 0.007), 500 × 10^3^ (*p* = 0.025), 1 × 10^6^ (*p* = 0.008), and 1.5 × 10^6^ (*p* < 0.0001) cell/mL. However, the difference was not significant compared to the seeding density of 125 × 10^3^ cell/mL (Fig. 3).

### The effect of bead-to-cell ratio on T cell expansion

**Fig. 4:**
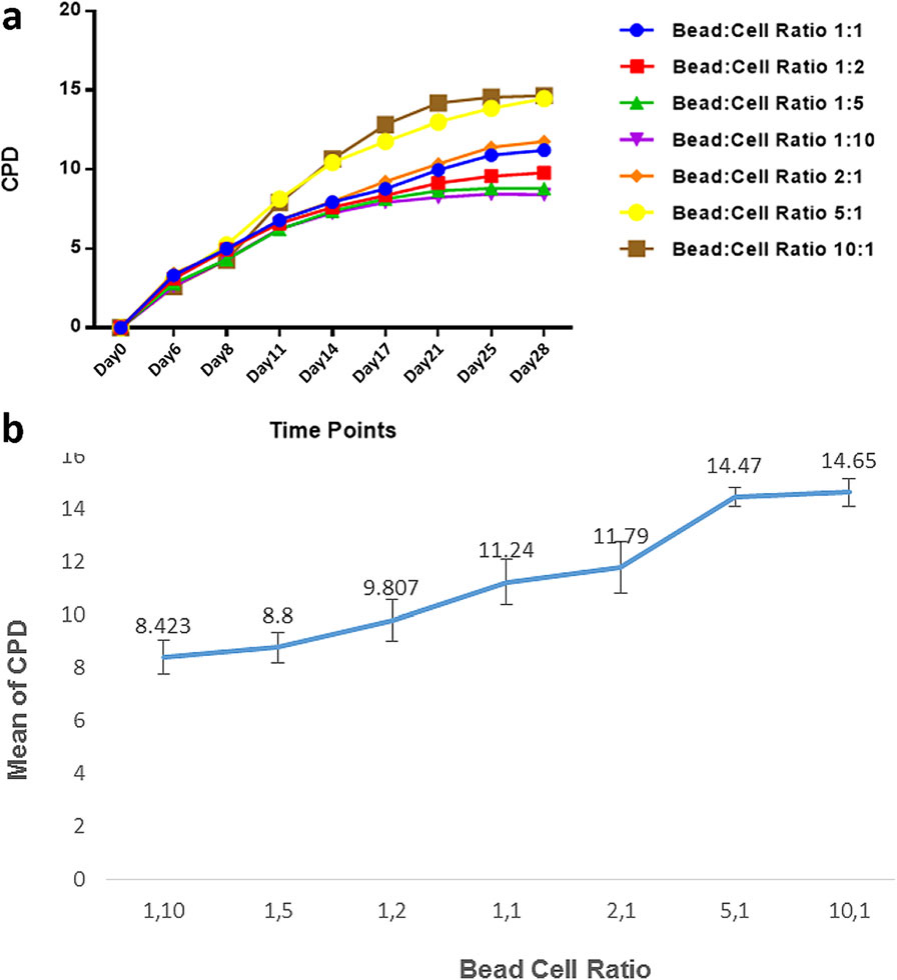
CPD results of T cells in various B:C ratio groups. Over the culture period, the highest B:C ratios generated the best expansion results (**a**). On day 28, a direct correlation between the number of beads and the expansion potential was seen (**b**)

T cell expansion showed a direct correlation to the B:C ratio. Therefore, the 10:1 B:C ratio and 1:10 B:C ratio resulted in the highest and lowest fold expansions at the ninth time point. On day 28, the differences between fold expansions of 1:10 and 1:5 (*p* = 0.9), 1:5 and 1:2 (*p* = 0.9), 1:2 and 1:1 (*p* = 0.9), 1:1 and 2:1 (*p* = 0.95) groups were not significant. The cells stimulated with a 5:1 B:C ratio resulted in significantly more fold expansions compared with those stimulated with 2:1 (*p* < 0.0001). Stimulation with a 10:1 B:C ratio provided no significantly higher expansion rate than stimulation with 5:1 (*p* = 0.087) (Fig. 4).

### Comparison of expansion rates in three donors

**Fig. 5:**
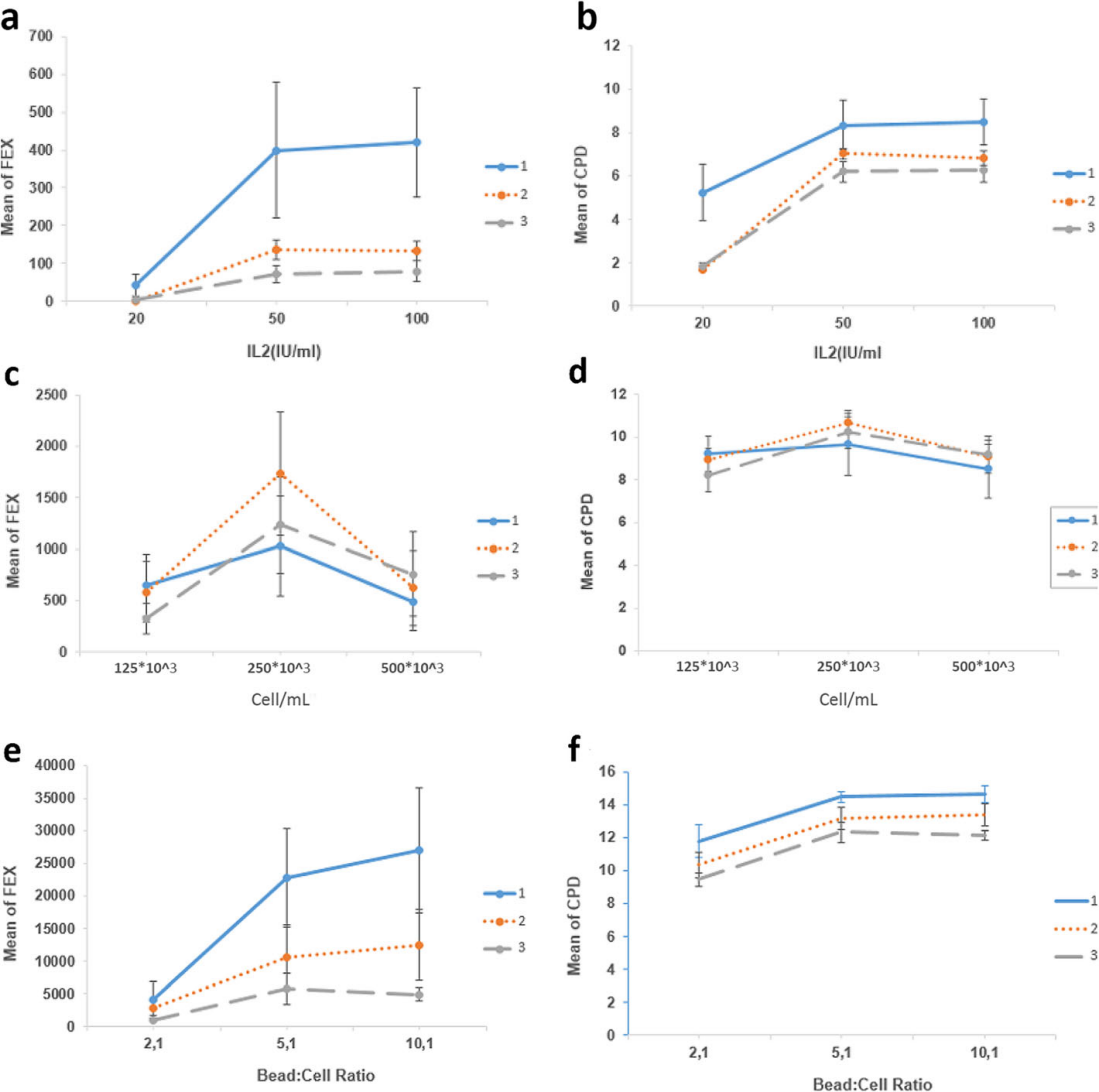
The fold expansion (**a, c, e**) and CPD (**b, d, f**) results of the three donors on day 28. Twenty international units per milliliter produced the lowest number of T cells, while the difference between 50 and 100 IU/mL was not significant (**a, b**). All samples experienced peak expansion in 250 × 10^3^ cell/mL (**c, d**). 2:1 B:C ratio results were lower than the other two, and while 10:1 increased expansion more than 5:1, the difference was not significant (**e, f**)

In order to determine the reproducibility of results, the PBMCs of three donors in the condition with the highest yield of expansion plus one level higher and one level lower as per each determinant factor were cultured, and their results were compared with each other. When comparing IL-2 concentrations, 20 IU/mL IL-2 was found to be the least potent concentration for T cell expansion in all three donors (Fig. 5a, b). In donors 1 and 3, 100 IU/mL IL-2 expanded more T cells compared to 50 IU/mL IL-2, and conversely, 50 IU/mL showed slightly more expansion potential than 100 IU/mL in donor 2, but none of these differences were statistically significant. Comparing the T cell expansions of the three donors according to seeding densities of 125 × 10^3^, 250 × 10^3^, and 500 × 10^3^ cell/mL, 250 × 10^3^ cells/mL seeding resulted in higher expansion rates (*p* < 0.0001) (Fig. 5c, d). Finally, expansions of PBMC of three donors using 2:1, 5:1, and 10:1 B:C ratio showed that 2:1 ratio led to the lowest expansion rate and there was no difference between 5:1 and 10:1 ratio in the donors (Fig. 5e, f).

### Immunophenotyping results

Immunophenotypic analysis showed the presence of 56–57% CD3^+^ cells in the PBMCs prior to expansion at day 0 which, after 28 days of expansion in the optimum condition, this number increased to approximately 96% in all donors (Table 1). Moreover, the rate of CD3/CD4^+^ cells decreased from about 27% at day 0 to 16% at day 28, and the majority of cells in the final time-point were CD3/CD8^+^.

**Table 1:** Flow cytometry profile of PBMCs (day 0) and T cells (day 28) of the three donors. The results are shown as mean ± SD

	CD3 (%)	CD4 (%)	CD8 (%)	CD3/CD4 (%)	CD3/CD8 (%)	CD4/CD8 (%)
**PBMC**						
Donor 1	57 ± 5.7	31.7 ± 2.4	26 ± 2.1	30.9 ± 6.4	21 ± 2	1.2 ± 0.5
Donor 2	56.9 ± 2.4	30.2 ± 2.6	26.9 ± 1.6	27.1 ± 6.1	24.5 ± 1.8	1.7 ± 0.7
Donor 3	56.8 ± 8.5	28.5 ± 4.3	27.4 ± 2.5	26.8 ± 12.8	25.6 ± 2.2	1.1 ± 0.2
**T cell**						
Donor 1	97.5 ± 1.8	19.3 ± 10.5	72.1 ± 19.4	17.3 ± 7.3	70.7 ± 13.4	1.34 ± 0.4
Donor 2	96.6 ± 1.9	16.5 ± 9.9	70.4 ± 15.8	14.7 ± 8.7	65.8 ± 11.5	2.1 ± 0.3
Donor 3	98 ± 1.3	20.4 ± 11.5	69.9 ± 17.7	16.7 ± 9.1	68.7 ± 10.3	1.5 ± 0.5

## Discussion

T-cells have a pivotal role in cellular immunity against tumor cells. One effective approach to cancer treatment would be a method for the isolation and ex vivo expansion of T cells. This requires optimized and highly efficient expansion protocols. To this end, isolated T-cells are first activated and then expanded in a well-defined condition to achieve a great number of cells. However, like many other cells, polyclonal T cell expansion lacks a unanimous efficient protocol [17]. In this study, we tried to optimize the expansion of polyclonal T cells in terms of three important determinants in the expansion of T cells: B:C ratio, IL-2 concentrations, and initial seeding density. We found that cultures in the condition of 250 × 10^3^ cells/mL seeding density in a 24-well plate activated with 5:1 B:C ratio and 50 IU/mL IL-2 supplementation provide the most optimal T cell expansion.

IL-2 is a potent cytokine that drives both T cell expansion and differentiation from naive phenotype to stem cell memory T cells (T_SCM_), central memory T cells (T_CM_), effector memory T cells (T_EM_), and finally terminally differentiated effector T cells (T_Eff_). T_Eff_ exhibit higher cytotoxic function and homing to peripheral tissues, while T_SCM_ show less senescence and great self-renewal and persistence [18]. Using other y-chain cytokines such as IL-7 and IL-15 have been proposed to augment T cell expansion and decrease differentiation. Although IL-7/IL-15-treated polyclonal T cells have less differentiation and more anti-tumor efficiency, they either show no significant T cell [19] expansion advantage or a small T cell [20] and CAR T cell expansion [21, 22] compared to IL-2 only-treated cells. Kaartinen and colleagues have reported that low IL-2 concentrations (0–5 IU/mL) while displaying meager T cell expansion, favor the less differentiated memory T cells, while higher concentrations (up to 300 IU/mL) produce T_Eff_ populations [16]. They also reported that the T_SCM_ subset was undetectable regardless of IL-2 concentration. Due to the need for high T cell numbers in clinical settings, the trials opt to use all CD3^+^ populations of T cells for CAR T cell or TCR T cell therapy [23–25]. A study in 2002 assessed the expansion of T cells from four donors while ranging IL-2 concentration from 0.2 to 2000 IU/mL. T cells showed a meager expansion at 0.2–20 IU/mL IL-2, as they expanded less than 10 folds. At 100–2000 IU/mL, T cells showed significant expansion compared to lower concentrations, but there was no difference between these groups [26]. Our results showed a higher expansion rate when increasing the IL-2 concentration from 20 to 50 IU/mL, but no significant difference in expansion rate occurred when IL-2 concentrations were further increased. This can be seen as an advantage, since the use of lower IL-2 concentrations not only reduces the costs of immunotherapies but also reduces negative IL-2 withdrawal effects on T cells post-expansion, diminishing T cell exhaustion [27, 28]. Moreover, in high IL-2 concentrations, endogenous IL-2 secretion is suppressed by Stat-5 and Blimp-1 proteins in negative feedback [29]. In a study T cells from three donors were expanded at three different concentrations of 20, 100, and 1000 IU/mL of IL-2 for 3 weeks. No significant difference until day 14 was observed; however, on day 21, the 100 IU/mL group showed the highest expansion rate, whereas the 20 IU/mL group resulted in the least expansion. Interestingly, 20 and 100 IU/mL concentrations yielded a 98% CD3 population, while 1000 IU/mL produced only 66% CD3+ cells [30]. In a similar study, Berglund et al. did not find any difference in T cell expansion over a one-week period when IL-2 ranged from 50 to 600 IU/mL [19]. Our findings corroborate these studies.

In all cell expansion protocols, seeding density plays an essential role, as it directly affects cell-cell, and in the case of T cell, cell-APC interactions and cytokine exchanges. Based on our findings, too high and too low densities are counterproductive, and equilibrium in cell/area is key. As we found, for T cell expansion, 250 × 10^3^ cells/mL of a 24-well plate is the optimum seeding density. Considering the unique surface area of the wells in a culture plate, the number of wells in each plate may show different results. Ma et al. compared the outcomes of T cell expansion using 1 × 10^6^, 1 × 10^5^, 1 × 10^4^, 1 × 10^3^ cell/mL densities in 6-well culture plates in 2 mL of medium and concluded that 1 × 10^6^ cell/mL (1 × 10^7^ cell/cm^2^) density produced the best results. The wells in 6-well and 24-well plates have 9.6 and 1.9 cm^2^ surface area, respectively. This means that 1 × 10^7^ cell/cm^2^ in a 6-well plate is approximately equivalent to 200 × 10^4^ cell/cm^2^ (200 × 10^3^ cell/mL) in a 24-well plate, which is closest to our results [31].

Anti-CD3/CD28 coated beads can act as artificial APCs, and so, are indispensable for T cell activation and subsequent expansion. To reveal the optimal B:C ratio with the highest expansion yield, Trickette et al. expanded T cells of four healthy donors at different B:C ratios for 2 weeks. They reported the least expansion in the 1:10 B:C ratio and an increased expansion when increasing the number of beads to 5:1 ratio, which was corroborated by our results [26]. In addition, they found that after expansion, the CD4^+^ population diminished to less than 15%, and CD8^+^ T cells constituted the majority of cells, which were also in agreement with our results. Shi et al. compared the expansion potential of 1:5, 1:1, and 3:1 B:C ratio over a 1-week period and reported the best expansion rate in the highest ratio, i.e., 3:1 ratio, and the least expansion in 1:5 [20]. Moreover, in the 1:5 B:C ratio, the CD3^+^ population consisted of equal portions of CD4^+^ and CD8^+^ cells, which could be due to poor cell expansion. When cells are activated, they require time to undergo the growth phases; hence, we opted to expand T cells for 4 weeks until they reach a plateau phase and compared the results [32]. This is evident in our results when expansion dwindles after 2–3 weeks, which is likely caused by T cells’ exhaustion and senescence. Therefore, to prevent ex vivo T cell exhaustion, most protocols opt to expand T cells for less than 2 weeks, because T cells typically lose function after 2 weeks of culture. The function of T cells could have been evaluated in our study by cytotoxicity assays (e.g. LDH assay) to ensure the production of functional T cells.

A step-up from small-scale static culture (e.g. culture plates and flasks) to large-scale T cell expansion would be to use bioreactors to mimic the in vivo environment in which T cells can rapidly proliferate. Although culture plates and flasks are the most traditional culture systems and are considered the gold standard of T cell culture, their efficacy is hindered by limited oxygen transfer, waste and lactate buildup, contamination risk, and lack of agitation. Novel, automated systems are seeking to rectify these issues. For instance, gas-permeable bags allow for a closed, sterile system with low media evaporation and efficient gas exchange but little control over culture parameters [33]. Currently, many commercial bioreactors such as G-REX® systems, a variety of rocking-motion bioreactors, CliniMACS® Prodigy, etc. are created to address the need for large-scale, clinical settings [34].

## Conclusion

The optimized culture conditions of T cell expansion are elusive since many different factors contribute to its outcome. Notably, T cell stimulation and co-stimulation methods, various cytokines, growth media and sera, oxygen concentration, ambient pH, initial seeding density, and static or dynamic culture are all elements that affect the result of T cell expansion. Here, we attempted to optimize the most prominent and base T cell culture conditions in laboratory settings. By comprehensive examination of these parameters, we concluded that 250 × 10^3^ cell/mL, 50 IU/mL IL-2, and 5:1 B:C ratio for initial activation is the most efficient and ideal condition among these three factors for the expansion of polyclonal T cells.

## Methods

### PBMC isolation and general T cell culture

This study was approved by the National Committee for Ethics in Biomedical Research (IR.TUMS.SPH.REC.1397.050). Peripheral blood was drawn from healthy donors after obtaining written informed consent. Before blood collection, the donors were clinically evaluated by a physician, and a complete blood cell count was conducted (Table 2). All donors were men and within a close range of age. The mononuclear cell portion of the peripheral blood (PBMC) was isolated using a density-gradient separation medium (1.077 g/mL, Ficoll-Hypaque, GE Healthcare, UK). The cell pellet was seeded in a 24-well culture plate containing 2 mL TexMACS medium (Miltenyi Biotec, Germany) supplemented with 10% heat-inactivated FBS (Gibco, USA) or platelet lysate (SABZ, Iran), 1X penicillin/streptomycin (Bioidea, Iran), and recombinant human IL-2 (Miltenyi Biotec, Germany). For T cell expansion and activation, T Cell Activation/Expansion Kit (Miltenyi Biotec, Germany) containing anti-CD2, −CD3, and -CD28- coated MACSiBead™ particles was used at different B:C ratio depending on the test group, and bead were removed on day 7 using a MACSiMAG™ Separator to prevent autofluorescence and T cell over-activation.

**Table 2:** The baseline demographic and hematological characteristics of the donors

Donor	WBC count (10^3^/μl)	Lymphocyte count (10^3^/μl)	lymphocyte percentage (%)	Sex	Age (year)
1	5.4	1.7	31.2	M	26
2	4.4	2.2	50.6	M	25
3	6.1	1.9	31.6	M	28

### Study grouping

The following groups were selected based on previously published literature and expansion studies in our research laboratory [6, 8]. At first, five groups in which IL-2 concentration was the variable were assigned to optimize IL-2 concentration. Secondly, six varying seeding density groups were appointed to discern the highest expansion rate of cell density. Lastly, seven groups of B:C ratio were selected after the result of the previous two experiments.

#### IL-2 concentration comparative groups

To assess the effect of IL-2 concentration on T cell expansion, PBMCs were seeded at a standard concentration of 1 × 10^6^ cells/mL in a 24-well culture plate, and were then stimulated once with microbeads at 1:2 B:C ratio, followed by expansion using five different IL-2 concentrations (20, 50, 100, 200, 500 IU/mL).

#### Seeding density comparative groups

To assess the effect of seeding density on T cell expansion, PBMCs were seeded at 62 × 10^3^, 125 × 10^3^, 250 × 10^3^, 500 × 10^3^, 1 × 10^6^, and 1.5 × 10^6^ cell/mL, and then were stimulated once with microbeads at 1:2 B:C ratio, followed by expansion using 50 IU/mL IL-2 which was selected based on the results from IL-2 concentration comparative groups.

#### B:C ratio comparative groups

To assess the effect of B:C ratio on T cell expansion, PBMCs were seeded at a concentration of 250 × 10^3^ cells/mL in a 24-well culture plate (based on the results of seeding density comparative groups), and were then stimulated once with microbeads at different B:C ratios (1:10, 1:5, 1:2, 1:1, 2:1, 5:1, and 10:1), followed by expansion using 50 IU/mL IL-2 (based on IL-2 concentration comparative groups).

### Monitoring T cell proliferation

Throughout the culture process, the cells were counted at nine time points (i.e., days 0, 6, 8, 11, 14, 17, 21, 25, and 28), and cell number was determined via trypan blue exclusion dye. The kinetics of T cell expansion was assessed under different culture conditions by calculating the cumulative population doubling (CPD) and fold expansion. We determined fold expansion by dividing the final cell count by the initial cell number. Population doubling (PD) was calculated using the equation: PD = 3.32 (log N1-logN0), where N0 represents the number of initially seeded cells and N1 represents the number of harvested cells at each time-point, respectively. After counting the cells, a number of cells equal to the initial seeding density of their respective group were subcultured to a new well. The cells were cultured at 37 °C in a humidified atmosphere containing 5% CO_2_ for 28 days. In all the experiments, 24-well culture plates containing 2 mL of cell and culture media mixture were used. Every 3 days, half the culture medium was replaced with a fresh medium containing the same concentration of IL-2.

### Comparison of T cell expansion of three donors in optimal and near-optimal conditions

**Fig. 6:**
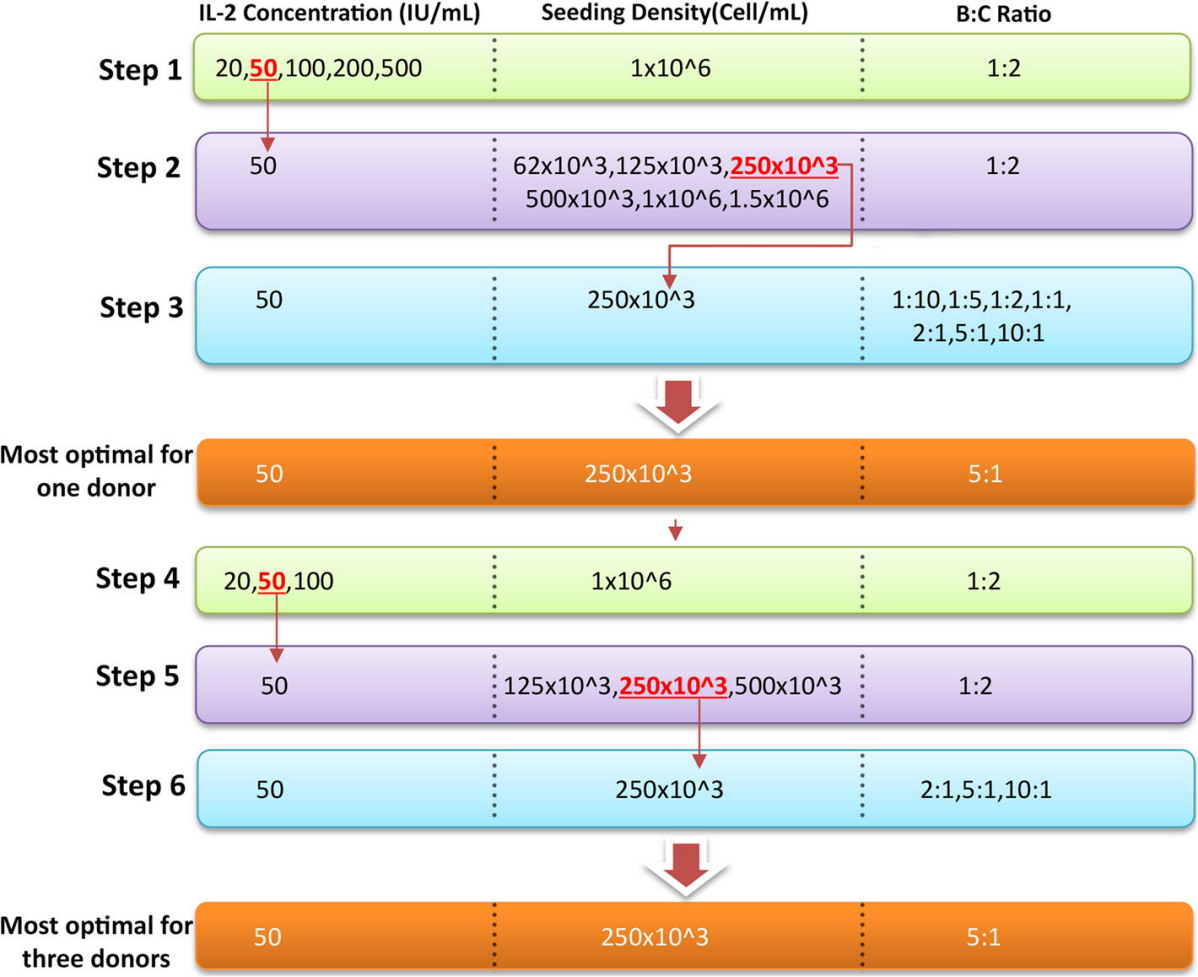
The schematic represents the steps to analyze and compare various T-cell culture conditions

After determining the best culture condition for the greatest yield of T cell in one donor, in each study group, PBMCs of three different donors, one of whom was the first donor, were expanded and compared in that condition to one level higher and one level lower. In other words, the PBMCs of the donors were seeded at 1 × 10^6^ cell/mL, stimulated with beads at 1:2 B:C ratio, and expanded using 20, 50, and 100 IU/mL IL-2. In the next experiment, the PBMCs of the donors were seeded at 125 × 10^3^, 250 × 10^3^, and 500 × 10^3^ cell/mL, stimulated with beads at 1:2 B:C ratio, and expanded using 50 IU/mL IL-2. Lastly, PBMCs of the three donors were seeded at 250 × 10^3^ cell/mL, stimulated with beads at 2:1, 5:1, and 10:1 B:C ratio, and expanded using 50 IU/mL IL-2 (Fig. 6).

### Flow cytometry analysis

Immunophenotyping was performed on PBMCs before expansion and T cells on day 28. Cells were washed in complete medium, and 2 × 10^5^ cells were re-suspended in the 100 μl PBS containing 3% Bovine Serum Albumin (Sigma, USA). Then. The cells were incubated with 5 μl of Anti-Human CD3-PerCP (Miltenyi Biotec, Germany), Anti-Human CD4-FITC/CD8-PE (BD Biosciences, USA), Mouse IgG2a Isotype PerCP (Miltenyi Biotec, Germany), Mouse IgG1 Isotype-FITC (BD Biosciences, USA), and Mouse IgG1 Isotype-PE Biosciences, USA) at 4 °C in the dark. Cells were analyzed with BD FACSCalibur™ Flowcytometer (BD Biosciences, USA) for cell surface markers and the data was processed using FlowJo software version X (FlowJo LLC, USA).

### Statistical analysis

The difference between cell expansion results was analyzed by Two-Way Repeated Measures ANOVA followed by Tukey post hoc test using GraphPad Prism version 8 for Windows. All the experiments were performed independently in triplicates. *P*-values less than 0.05 were considered statistically significant.

## Data Availability

The datasets used and/or analyzed during the current study are available from the corresponding author on reasonable request.
